# Coronary slow flow phenomenon: a meta-analysis of clinical risk predictors

**DOI:** 10.3389/fcvm.2026.1656151

**Published:** 2026-05-07

**Authors:** Danpeng Wang, Yanwei Li, Jinming Hu, Ke Zhang

**Affiliations:** 1 Department of Cardiology, The Second Affiliated Hospital of Chengdu University of Traditional Chinese Medicine, Chengdu, China; 2 Department of Cardiology, Hospital of Chengdu University of Traditional Chinese Medicine, Chengdu, China; 3 Department of Cardiology, Pengzhou Traditional Chinese Medicine Hospital, Pengzhou, China

**Keywords:** circulating biomarkers, clinical variables, coronary slow flow phenomenon, risk factors, systematic review and meta-analysis

## Abstract

**Background:**

As coronary angiography becomes more common, more cases of coronary slow flow phenomenon (CSFP) are detected. To identify key clinical predictors of CSFP, we conducted a meta-analysis. This aims to improve treatment decisions for affected patients.

**Methods:**

We searched multiple databases (Embase, Web of Science, PubMed) up to May 2025 for studies on the association between clinical risk factors and CSFP. Study heterogeneity was assessed using Cochran's *Q* test and I^2^ statistics, followed by meta-analysis to pool effect estimates. Publication bias was evaluated with funnel plots and Egger's test. All analyses were conducted using R.

**Results:**

We identified 23 eligible studies, comprising a total of 2,309 patients with CSFP and 3,377 controls. The pooled analysis identified several clinically independent risk factors for CSFP: Triglycerides (TG)[odds ratio (OR) = 1.01, 95% confidence interval (CI): 1.01–1.02)], Totol cholesterol (TC)[OR = 1.008, CI: 1.001–1.015],White blood cell (WBC) counts[OR = 1.07, CI: 1.04–1.10], Platelets/lymphocytes ratio (PLR)[OR = 1.01, CI: 1.01–1.02], Body mass index (BMI)[OR = 1.09, CI: 1.05–1.13], and platelet count (PC) [OR = 1.009, CI: 1.006–1.011], Current smoke (OR = 1.09, 95% CI = 1.07–1.10) were significantly associated with an increased risk of CSFP.

**Conclusion:**

This comprehensive meta-analysis identifies seven key modifiable risk factors for CSFP. These findings not only enhance risk prediction models but also suggest potential therapeutic targets through lipid optimization, anti-inflammatory, antiplatelet strategies, weight control and smoking cessation interventions in CSFP management.

**Systematic Review Registration:**

PROSPERO CRD420251057679.

## 1 Introduction

The coronary slow flow phenomenon (CSFP) is defined as angiographically evident delayed distal coronary opacification during selective coronary angiography, occurring in the absence of significant epicardial coronary artery stenosis (luminal diameter reduction <40%). First described by Tambe et al. in 1972 (1), Epidemiologic studies estimate the prevalence of CSFP at 1%–5% among patients undergoing coronary angiography for suspected ischemic heart disease, particularly in those without obstructive coronary artery disease (2). Affected individuals commonly present with anginal symptoms, including resting angina and exertional chest pain. While CSFP is associated with a low overall mortality rate (<1%), accumulating clinical evidence highlights its strong correlation with life-threatening ventricular arrhythmias (e.g., polymorphic ventricular tachycardia) and recurrent syncope (3, 4).

Pathophysiological substrate of myocardial ischemia, intricately linked to systemic endothelial dysfunction (5), chronic low-grade inflammation (6), and impaired coronary microvascular reserve capacity were involved in CSFP (7). Current therapeutic strategies prioritize statins and nicorandil to mitigate microvascular dysfunction and improve coronary perfusion (8, 9). Although cardiac magnetic resonance (CMR) and coronary microcirculatory resistance index (IMR) have been widely used, CMR assesses microcirculatory function semi-quantitatively through the myocardial perfusion reserve index (MPRI) but cannot directly measure microvascular resistance. Additionally, low temporal resolution and prolonged examination time increase patient discomfort and may introduce motion artifacts, reducing image quality (10, 11). Other studies have shown that coronary slow flow (CSF) has only a weak correlation with invasive microcirculatory function tests (such as CFR, IMR) (e.g., the correlation coefficient r = 0.16 for IMR) (12), and the critical value standards for different microcirculatory parameters (such as CFR < 2.0, IMR≥25, MRR < 2.1) are not uniform (13). Therefore, this study shifts toward integrating non-invasive examination factors related to CFSP.

Emerging evidence has shifted research focus toward multifactorial risk prediction models integrating circulating biomarkers, anatomic parameters (e.g., coronary artery calcium score), and clinical variables (e.g., smoking, diabetes mellitus) (14, 15). Given the established associations of CSFP with endothelial dysfunction, chronic low-grade inflammation, and impaired microvascular regulation, this study specifically focuses on the following categories of indicators that directly reflect or influence these key processes: (1)Inflammatory Activation Markers: Such as high-sensitivity C-reactive protein (hs-CRP) and white blood cell count, which directly quantify the chronic low-grade inflammatory state associated with CSFP; (2) Endothelial Function and Metabolic Indicators: Including lipid parameters such as total cholesterol and body mass index. These are not only traditional risk factors for atherosclerosis but also directly contribute to microvascular dysfunction by affecting endothelial nitric oxide bioavailability and promoting oxidative stress; (3) Hemorrhological and Prothrombotic State Indicators: Such as platelet count, where abnormal levels or activity may influence hemodynamics within the microcirculation and promote *in-situ* thrombus formation; (4) Established Behavioral and Clinical Risk Factors: Such as smoking, which can directly impair endothelial function and exacerbate systemic inflammation. By systematically analyzing these key measurable factors spanning inflammation, endothelium, metabolism, and hemodynamics, this study aims to establish a more pathophysiologically grounded risk assessment framework for CSFP. we performed a systematic review and meta-analysis to quantify the association between modifiable and non-modifiable risk factors and CSFP, aiming to inform targeted therapeutic interventions.

## 2 Methods

### 2.1 Search strategy

Based on the Preferred Reporting Items for Systematic Reviews and Meta Analysis Guidelines, was conducted across three major electronic databases (Embase, Web of Science, and PubMed) from their inception through May 2025. The Medical Subject Headings (MeSH) terms searched for the literature are as follows: ((coronary slow flow) OR (coronary slow flow phenomenon) OR (CSFP) OR (CSF)) AND ([risk factor(Title/Abstract)] OR [risk factors(Title/Abstract)]) OR (predictor factor) OR (influence factor)).

### 2.2 Literature screening and data extraction

Studies were selected based on the following methodological requirements: (1) Comparative clinical investigations evaluating human physiological parameters; (2) case-control studies or cohort studies with the Coronary Slow Flow Phenomenon (CSFP) as the exposure group and patients exhibiting normal coronary blood flow with luminal stenosis <40% as the control group; (3) availability of directly calculated or derivable odds ratios (ORs) with 95% confidence(95% CIs) intervals through reported data; (4) standardized quantification of coronary flow velocity using the validated Thrombolysis In Myocardial Infarction (TIMI) frame count methodology. The cutoff value for coronary slow flow is cTFC ≥25 or TIMI flow grade ≤2 (16).

Exclusion criteria for the study are as follows: (1) absence of CSFP as a primary or secondary investigation focus; (2) redundant datasets from overlapping study populations or duplicate publications; (3) Insufficient quantitative data for effect size calculation or outcome verification.

This meta-analysis aims to synthesize effect estimates from various original studies, adjusted for multivariate factors, to control for the influence of potential confounding factors. The effect measures we extracted primarily come from multivariate logistic regression models in the original studies. These models adjust for variables in the following categories: 1. circulating biomarkers: (1) inflammatory factors; (2) lipid indicators; (3) other indicators such as platelets; 2. clinical variables: diabetes, hypertension, BMI, smoking status.

Handling of confounding factors: 1) Obtain as many adjusted effect sizes as possible during the data extraction phase; 2) If the original study provides both unadjusted and adjusted data, prioritize extracting the multivariable-adjusted effect sizes.

Model assumption evaluation: For highly correlated variables such as sugar metabolism, we address the issue of multicollinearity by establishing extraction priorities (e.g., in sugar metabolism variables HbA1c, fasting glucose, and diabetes status, diabetes status is prioritized). We perform sensitivity analysis using the leave-one-out method. If removing a specific study leads to a significant change in the combined effect size, this indicates that the study may have unique characteristics (e.g., different model specifications, larger population differences, or data quality issues). We use Egger's test and funnel plots to assess publication bias and correct it through trimming and filling.

### 2.3 Literature quality assessment

The quality of the included studies was assessed using the modified Newcastle-Ottawa Scale (NOS), with studies scoring 6 or above considered high quality.

### 2.4 Statistical analysis

All statistical analyses were conducted using R statistical software (version 4.5.0; R Foundation for Statistical Computing). To quantify the association between clinical variables and CSFP, odds ratios (ORs) with corresponding 95% confidence intervals (CIs) were calculated. For the meta-analysis component, heterogeneity across studies was quantitatively assessed using the I^2^ statistic and Cochran's *Q* test. A fixed-effects model (Mantel-Haenszel method) was applied when heterogeneity was deemed non-significant (Cochran's *Q* test *p* ≥ 0.10 and I^2^ ≤ 50%), under the assumption that all included studies estimate a single common effect. In contrast, a random-effects model (DerSimonian-Laird method) was employed in the presence of significant heterogeneity (*p* < 0.10 or I^2^ > 50%) to account for variability beyond sampling error, thereby providing a more conservative and generalizable pooled estimate. sensitivity analysis used the leave-one-out method. Publication bias was assessed through Egger's regression asymmetry test and funnel plot, There was significant bias in Egger's t-test results (*p* < 0.05), which were adjusted using the trim and fill method to ensure the robustness of the outcomes.

Handling of continuous variables and data conversion: We simultaneously extracted descriptive statistics of continuous variables (mean ± standard deviation) and effect size data (OR values). The former was used to compare baseline differences between groups, and the latter was used to analyze their strength as risk factors. ORs were directly obtained from the literature. For each continuous variable, we determined a unique combination model based on the general format of the data before data synthesis.

## 3 Results

### 3.1 Literature search

**Figure 1:**
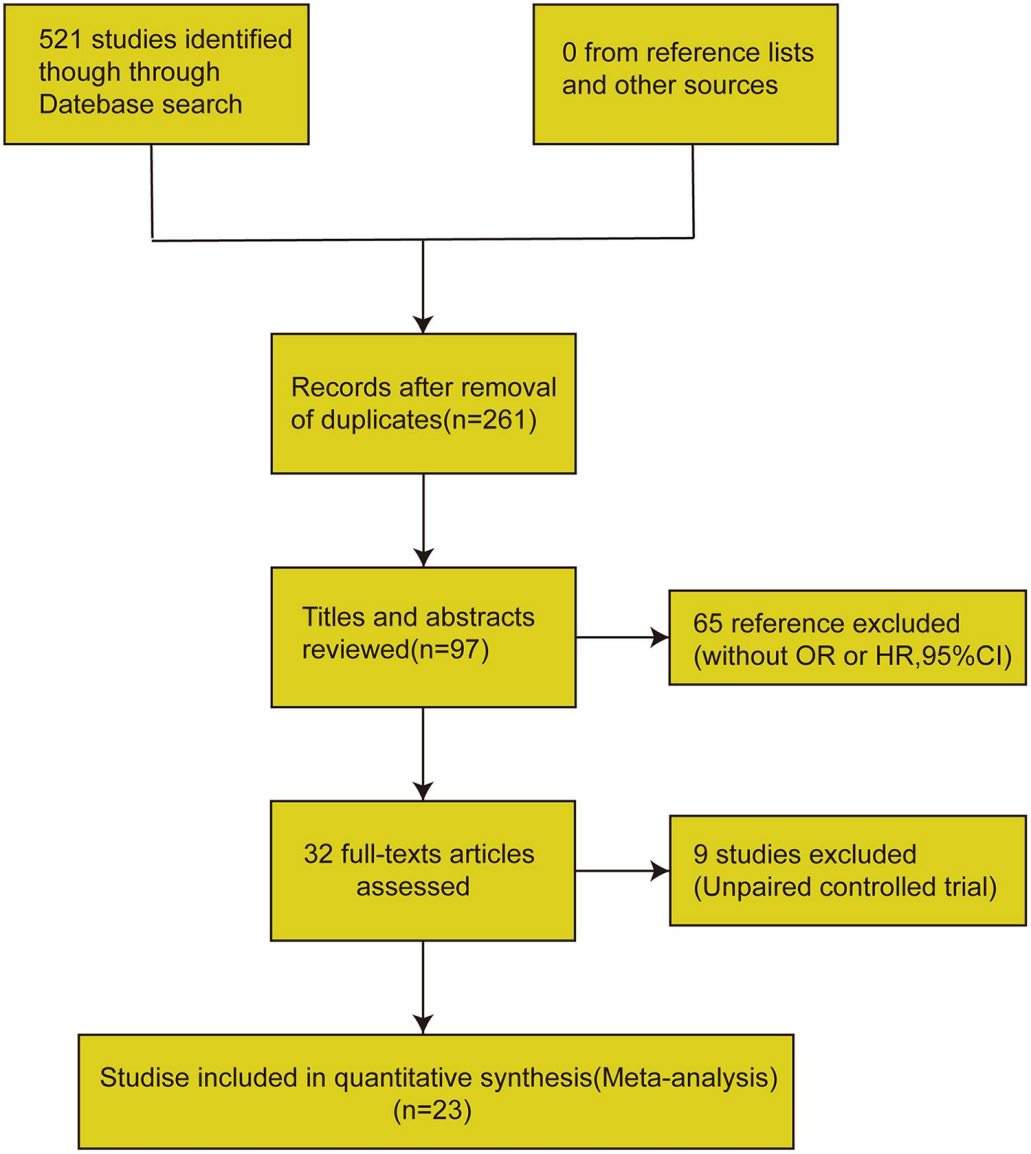
The screening flow chart.

The systematic search initially identified 521 potentially relevant publications. After the deletion of duplicate documents, 261 articles were retained. Through reading the titles and abstracts, 164 records were excluded for focusing on obstructive coronary artery disease (luminal stenosis ≥40%) or acute myocardial infarction. 65 publications were excluded due to insufficient statistical reporting ORs, risk ratios, or 95% CIs, 32 articles progressed to comprehensive review, with 9 excluded for protocol non-compliance. 23 articles meeting the inclusion criteria were included. The complete selection workflow is detailed in the PRISMA diagram (Figure 1).

**Figure 2:**
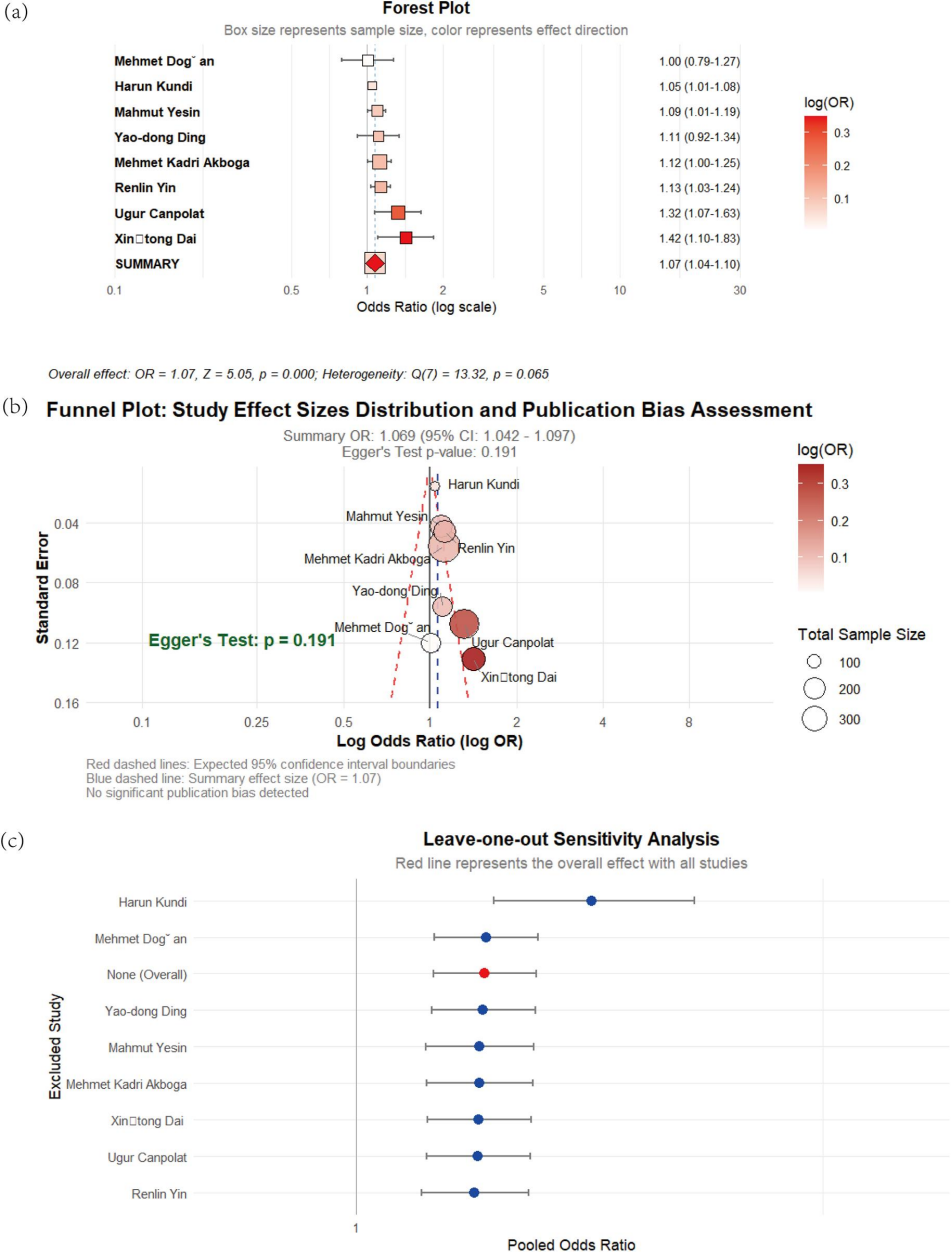
**(a)** Forest plot of the impact of WBC count on CSFP; **(b)** the funel plot; **(c)** sensitivity analysis.

### 3.2 Data characteristics of the included studies

The meta-analysis incorporated 23 case-control studies published between 2002 and 2025, comprising 2,309 patients with coronary slow flow phenomenon (CSFP) and 3,377 controls with angiographically confirmed normal coronary flow (luminal stenosis <40%). Geographically, the studies originated predominantly from Turkey (*n* = 12, 52.2%) and China (*n* = 10, 43.5%), with one U.S.-based study (4.3%). All investigations reported core demographic variables (age, gender) and clinical risk profiles, including smoking status and biomarker measurements. Systematic comparisons of these parameters between CSFP and control cohorts and NOS score are presented in Table 1.

**Table 1:** Basic information included in the study and NOS score.

study	Year	CSFP	NCF	country	Age [mean(SD)] CSFP/NCF		male (n/%) CSFP/NCF		Statin therapy, n (%) CSFP/NCF		Study design	NOS score
Taner Ucgun (17)	2014	45	55	Turkey	59.4 (9.2)	57.8 (9.3)	33 (70)	30 (54)	5 (11.1)	5 (9.1%)	CC	6
Fahrettin Oz1 (18)	2015	22	200	Turkey	61 (9.16)	59.7 (7.54)	14 (65)	124 (62)	3 (13.6)	32 (16)	CC	6
Ming-fang Ye (19)	2016	93	206	China	59.55 (8.69)	58.59 (9.85)	57 (61.29)	123 (59.71)	—	—	CC	6
Mehmet Kadri Akboga (20)	2015	221	293	Turkey	53.8 (10.4)	53.3 (9.6)	158 (71.5)	193 (65.9)	—	—	CC	5
Mehmet Dogan (21)	2013	82	98	Turkey	51.5 (8.6)	49.7 (11.9)	68 (82.9)	81 (82.7)	—	—	CC	5
Ugur Canpolat (22)	2015	253	176	Turkey	53.7 (9.3)	55.1 (9.1)	158 (62.4)	112 (63.6)	35 (13.8)	15 (8.5)	CC	6
Harun Kundi, MD (23)	2015	53	35	Turkey	59 (15)	56 (13)	34 (64.1)	22 (62.8)	—	—	CC	5
Mustafa etin (24)	2016	78	50	Turkey	52.7 (10)	51.5 (8.1)	49 (62.8)	31 (62)	18 (23.1)	19 (38)	CC	5
Yasin Yuksel (25)	2023	50	40	Turkey	—	—	80 (60.3)	75 (65.2)	—	—	CC	5
Beau M. Hawkins (2)	2012	96	62	USA	54.5 (10.0)	56.4 (9.3)	48 (77.8)	85 (88.5)	39 (63.5)	58 (60.4)	CC	5
Lijun Han (26)	2024	69	586	China	—	—	49 (71.01)	382 (65.19)	—	—	CC	6
Jiang Yu (14)	2024	435	294	China	56 (10)	58 (11)	277 (63.7)	118 (40.1)	—	—	Cohort	6
Tao Wang (27)	2016	76	108	China	58.57 (10.18)	56.44 (10.25)	49 (64.47)	60 (55.56)	17 (22.37)	16 (14.81)	CC	6
ShuWen Zang (28)	2024	85	170	China	55.5 (11.9)	55.5 (11.9)	51 (60.0)	102 (60.0)	62 (72.9)	120 (70.6)	CC	7
Huseyin Celebi (29)	2007	48	29	Turkey	53.4 (9.5)	53.5 (8.5)	21 (43.7)	5 (17.2)	14 (29.2)	10 (34.5)	CC	5
Tahir Durma (30)	2013	45	45	Turkey	51.7 (7.1)	50.4 (7.2)	14 (31.1)	13 (28.9)	4 (8.9%)	5 (11.1)	Cohort	6
Mahmut Yesin (31)	2019	81	136	Turkey	67.2 (12.7)	65.4 (11.6)	38 (46.9)	86 (63.2)	—	—	Cohort	6
Gonul Aciksari (32)	2021	84	83	Turkey	54.6 (8.9)	55.2 (8.5)	52 (61.9)	41 (49.4)	12 (14.3)	16 (19.3)	Cohort	6
Yaodong Ding (33)	2020	78	92	China	60.2 (9.7)	62.7 (9.5)	55 (70.5)	48 (52.1)	33 (42.8%)	33 (35.9)	CC	7
Xintong Dai (34)	2022	89	167	China	59.6 (5.6)	58.5 (6.3)	56 (62.9)	90 (53.9)	20 (22.5)	39 (23.4)	CC	7
Xiaojiao Zhang (35)	2024	79	158	China	61.2 (9.6)	61.2 (9.6)	61 (77.2)	118 (74.7)	28 (35.4)	52 (32.9)	Cohort	7
ZhiGao Wen (36)	2024	71	142	China	52.6 (10.8)	52.6 (10.8)	50 (70.4)	100 (70.4)	26 (36.6)	45 (31.7)	CC	7
Renlin Yin (37)	2025	76	152	China	57.29 (13.11)	57.29 (13.07)	44 (57.9)	117 (77)	22 (28.9)	50 (32.9)	CC	6

### 3.3 Main results of meta-analysis

#### 3.3.1 Inflammatory indicators

##### 3.3.1.1 White blood cell (WBC) count

A total of eight observational studies examined the relationship between WBC counts and CSFP risk. Given the absence of significant heterogeneity across studies (I^2^ = 47.5%), we employed a fixed-effects model for pooled analysis. The meta-analysis demonstrated a significant association between elevated WBC counts and CSFP risk, with a pooled OR of 1.07 (95% CI: 1.04–1.10, *p* < 0.01). However, Egger's regression test showed no evidence of publication bias in the analysis (*p* = 0.191), as visually illustrated in Figure 2.

##### 3.3.1.2 HsCRP

Four prospective cohort studies evaluated the association between hsCRP levels and CSFP risk. The pooled analysis using a fixed-effects model (I^2^ = 48.4%, indicating nonsignificant heterogeneity) demonstrated statistically significant association between elevated hsCRP levels and CSFP risk, with a pooled OR of 1.01 (95% CI: 1.00–1.01, *p* < 0.01). Notably, Egger's regression test showed no evidence of publication bias in the analysisEgger's regression test detected substantial publication bias in the available evidence (*p* = 0.009), as visually illustrated in Supplementary Figure S1.

##### 3.3.1.3 Platelets/lymphocytes ratio (PLR)

**Figure 3:**
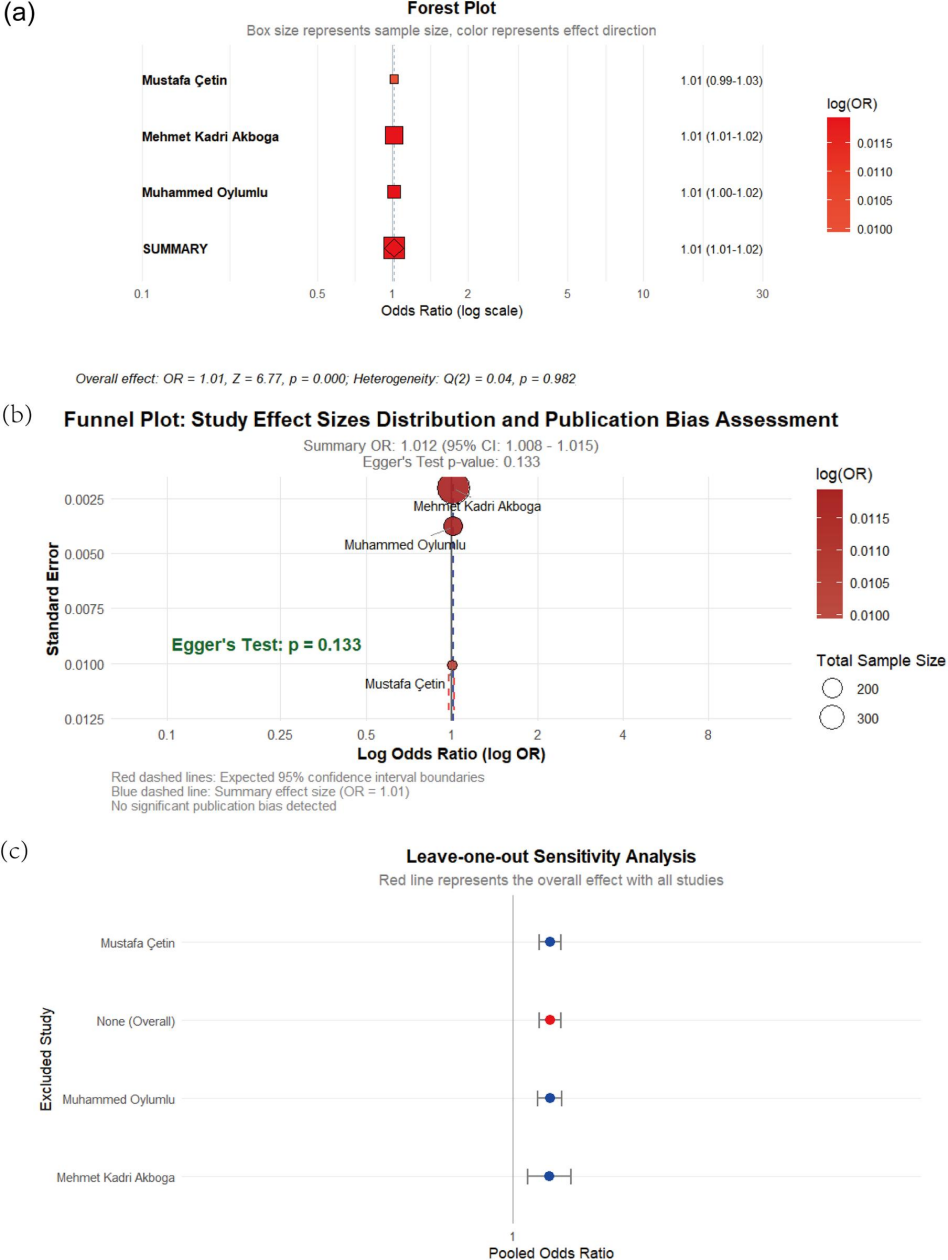
**(a)** Forest of plot of the impact of PLR on CSFP; **(b)** the funel plot; **(c)** sensitivity analysis.

Three case-control studies examined the relationship between PLR and CSFP risk. The meta-analysis demonstrated negligible heterogeneity among studies (I^2^ = 0%), supporting the application of a fixed-effects model. Pooled analysis revealed statistically significant association between PLR elevation and CSFP risk (OR: 1.01, 95% CI: 1.01–1.02, *p* < 0.01). Egger's regression test showed no evidence of publication bias in the analysis (*p* = 0.133), as visually illustrated in Figure 3.

#### 3.3.2 Lipid-related indicators

##### 3.3.2.1 High-density lipoprotein cholesterol (HDL)

Eleven observational studies evaluated the relationship between high-density lipoprotein (HDL) levels and the risk of coronary slow flow phenomenon (CSFP). A random-effects meta-analysis (I^2^ = 53.5%) demonstrated a significant inverse association between higher HDL levels and CSFP risk, with a pooled odds ratio (OR) of 0.60 [95% confidence interval (CI): 0.46–0.76, *p* < 0.01]. Significant publication bias was detected by Egger's regression test (*p* < 0.0001). Subsequent trim-and-fill analysis indicated no significant association after adjustment for potential missing studies (OR = 0.99, 95% CI: 0.96–1.02, *p* = 0.34), as presented in Supplementary Figure S2.

##### 3.3.2.2 Low-density lipoprotein cholesterol (LDL)

Seven observational studies evaluated the relationship between LDL cholesterol levels and CSFP risk. Significant heterogeneity was observed among the included studies (I^2^ = 59.4%), necessitating the use of a random-effects model. The pooled analysis demonstrated no statistically significant association between elevated LDL levels and CSFP risk (OR = 0.99, 95% CI: 0.97–1.02, *p* = 0.547). Egger's regression test indicated no evidence of publication bias (*p* = 0.097), as visually illustrated in Supplementary Figure S3.

##### 3.3.2.3 Totol cholesterol(TC)

**Figure 4:**
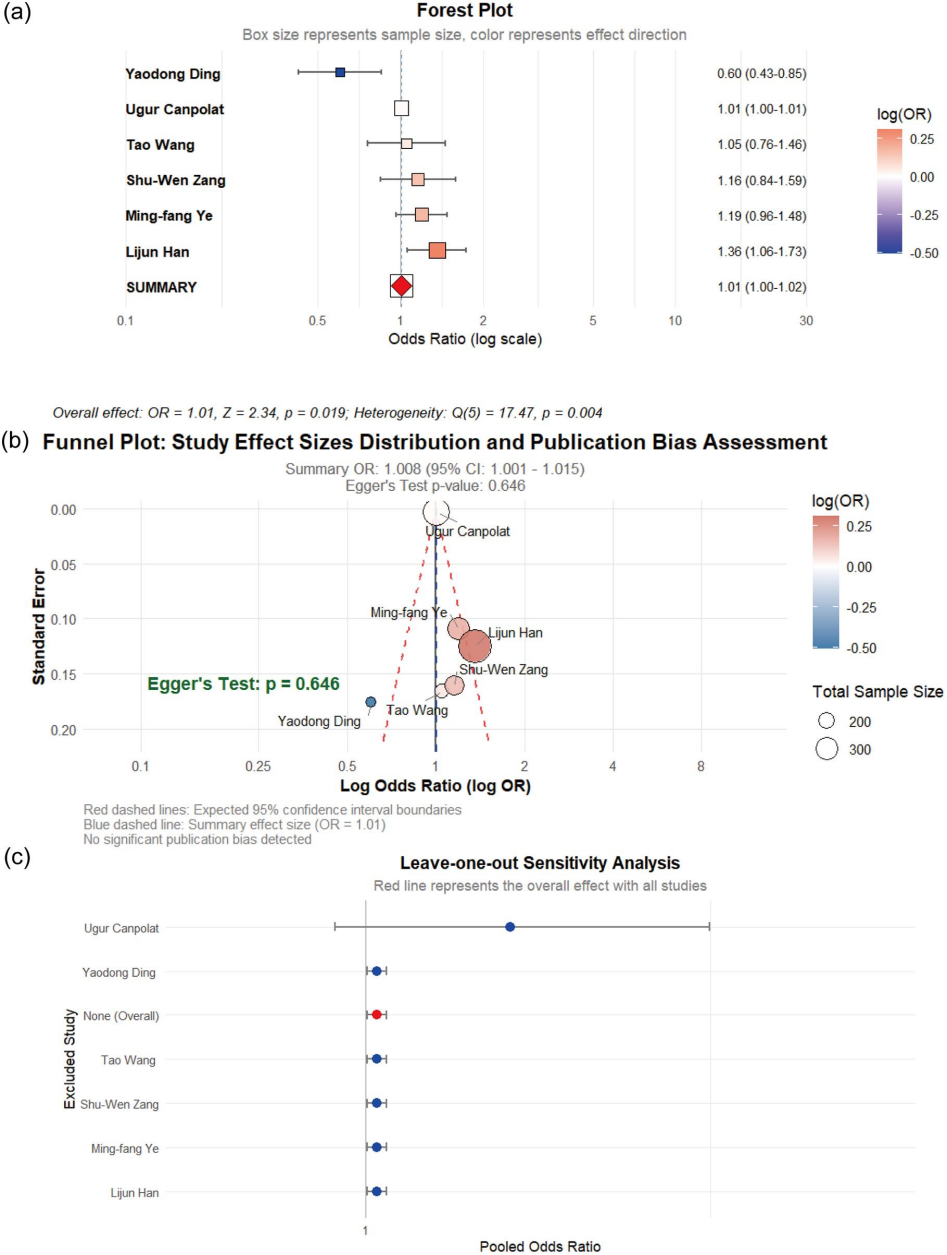
**(a)** Forest plot of the impact of TC on CSFP; **(b)** the funel plot; **(c)** sensitiity analysis.

Six observational studies examining the association between TC levels and CSFP risk were included in this meta-analysis. Substantial heterogeneity was identified across studies (I^2^ = 71.4%), prompting the random-effects model. The pooled analysis revealed a statistically significant but clinically marginal association between elevated PC and CSFP risk (OR = 1.009, 95% CI: 1.006–1.011, *p* < 0.01). However, Egger's regression test showed no evidence of publication bias (*p* = 0.646), as visually illustrated in Figure 4.

##### 3.3.2.4 Triglycerides(TG)

**Figure 5:**
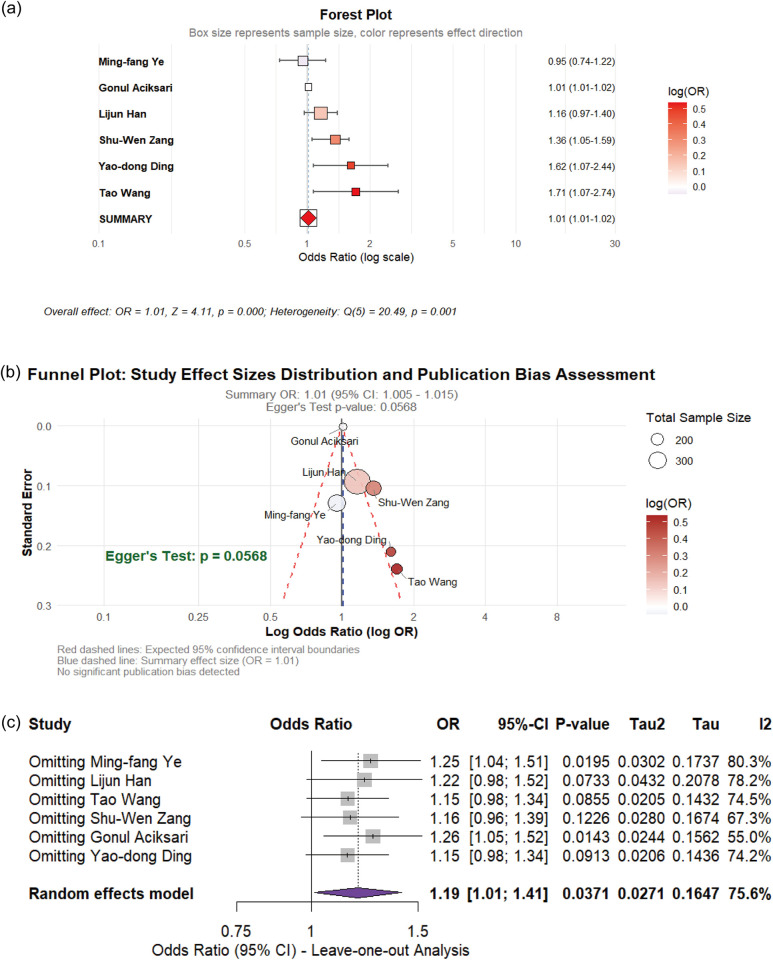
**(a)** Forest plot of the impact of TG on CSFP; **(b)** the funel plot; **(c)** sensitivity analysis.

Six cohort studies evaluated the relationship between elevated TG levels and CSFP risk. The meta-analysis revealed substantial heterogeneity among included studies (I^2^= 75.6%), the random-effects model was applied. Using a corrected square root weighting method, the pooled analysis demonstrated a statistically significant positive association between TG elevation and CSFP risk (OR = 1.01, 95% CI: 1.01–1.02, *p* < 0.01). Egger's regression test indicated significant publication bias (*p* = 0.057)(see Figure 5).

#### 3.3.3 Comorbidities

##### 3.3.3.1 Platelet count (PC)

**Figure 6:**
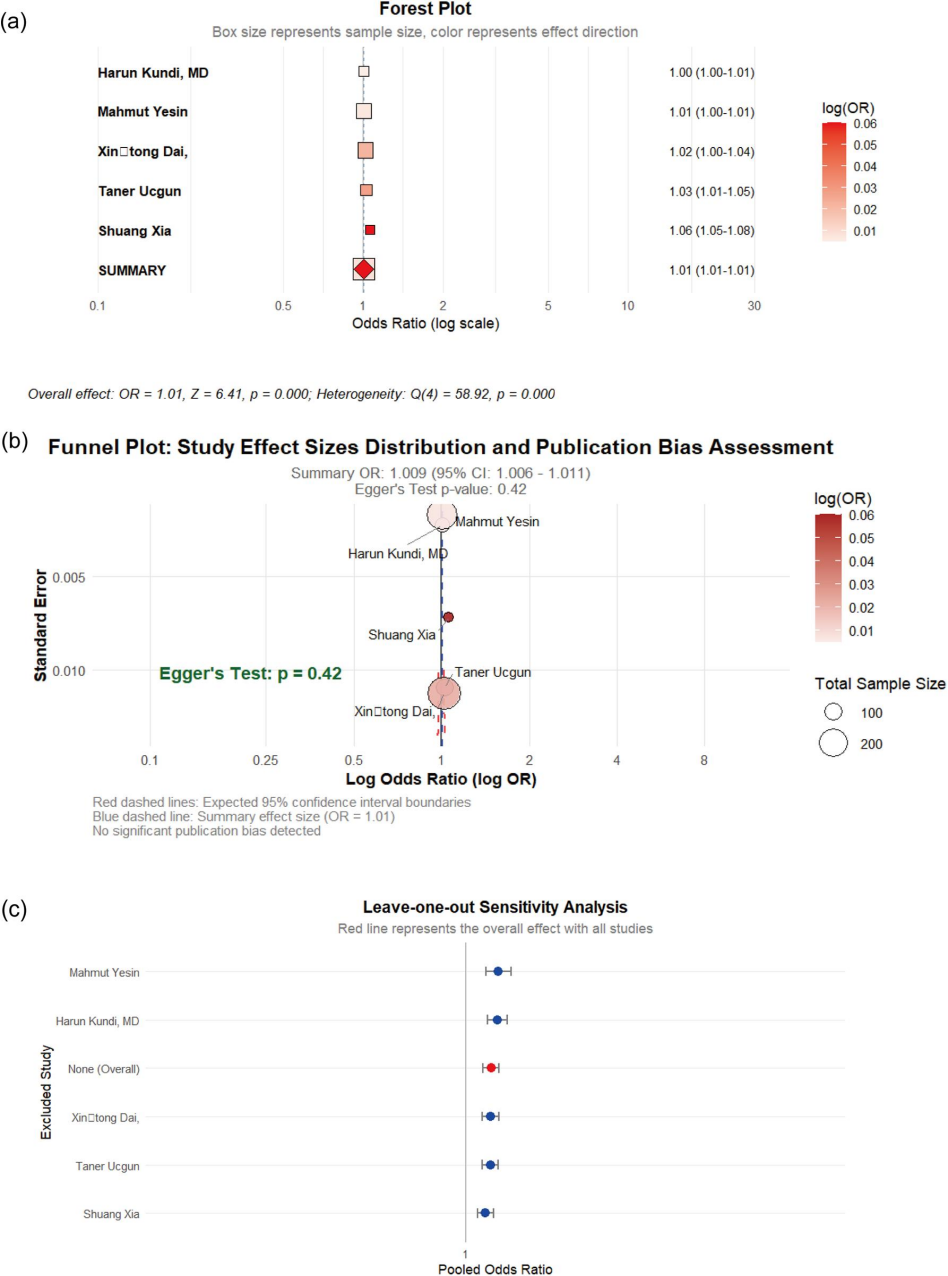
**(a)** Forst plot of the impact of PLT on CSFP; **(b)** the funel plot; **(c)** sensitivity analysis.

Five case-control studies evaluated the association between platelet counts and CSFP)risk. The meta-analysis demonstrated extreme heterogeneity among included studies (I^2^= 93.2%), prompting the application of a random-effects model. The pooled analysis revealed a statistically significant but clinically marginal association between elevated PC and CSFP risk (OR = 1.009, 95% CI: 1.006–1.011, *p* < 0.01). Egger's regression test indicated no statistically significant publication bias (*p* = 0.42), (see Figure 6).

##### 3.3.3.2 Hypertension

Eleven observational studies evaluated the relationship between **Hypertension** and the risk of coronary slow flow phenomenon (CSFP). A random-effects meta-analysis (I^2^ = 57%) demonstrated a significant inverse association between higher HDL levels and CSFP risk, with a pooled odds ratio (OR) of 1.38 [95% confidence interval (CI): 1.16–1.69, *p* < 0.01]. Significant publication bias was detected by Egger's regression test (*p* = 0.045). Subsequent trim-and-fill analysis indicated no significant association after adjustment for potential missing studies (OR = 0.97, 95% CI: 0.94–1.00, *p* = 0.34), as presented in Supplementary Figure S4.

##### 3.3.3.3 Diabetes(DM)

Ten cohort studies evaluated the association between DM and CSFP risk. The meta-analysis demonstrated significant heterogeneity across studies (I^2^= 62.2%), leading to the application of a random-effects model. Pooled analysis demonstrated statistically significant association between elevated DM and CSFP risk (OR = 1.16, 95% CI: 1.03–1.30, *p* = 0.016). Significant publication bias was detected by Egger's regression test (*p* = 0.019). Subsequent trim-and fill analysis indicated no significant association after adjustment for potential missing studies (OR = 1.15, 95% CI: 0.89–1.47, *p* = 0.28), as presented in Supplementary Figure S5.

##### 3.3.3.4 Body mass index (BMI)

**Figure 7:**
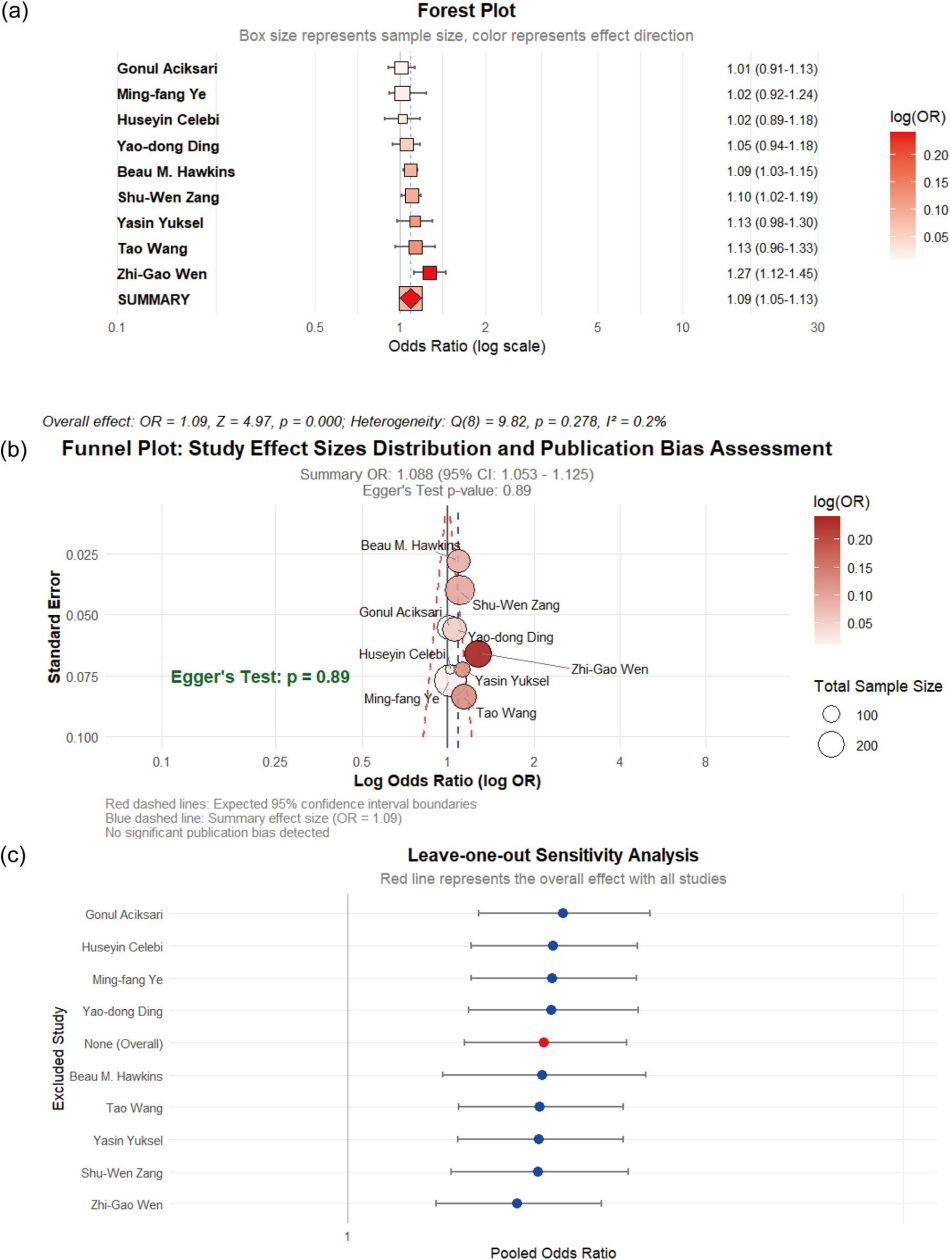
**(a)** Forest plot of the impact of BMI on CSFP; **(b)** the funel plot; **(c)** sensitivity analysis.

Eleven observational studies examined the association between BMI and CSFP risk. The meta-analysis demonstrated low heterogeneity among studies (I^2^= 56.3%), supporting the use of a random-effects model. Pooled analysis revealed a significant positive trend between elevated BMI and CSFP risk (OR = 1.09, 95% CI: 1.05–1.13, p < 0.01). Egger's regression test showed no evidence of publication bias (*p* = 0.890) (see Figure 7).

##### 3.3.3.5 Current smoke

**Figure 8:**
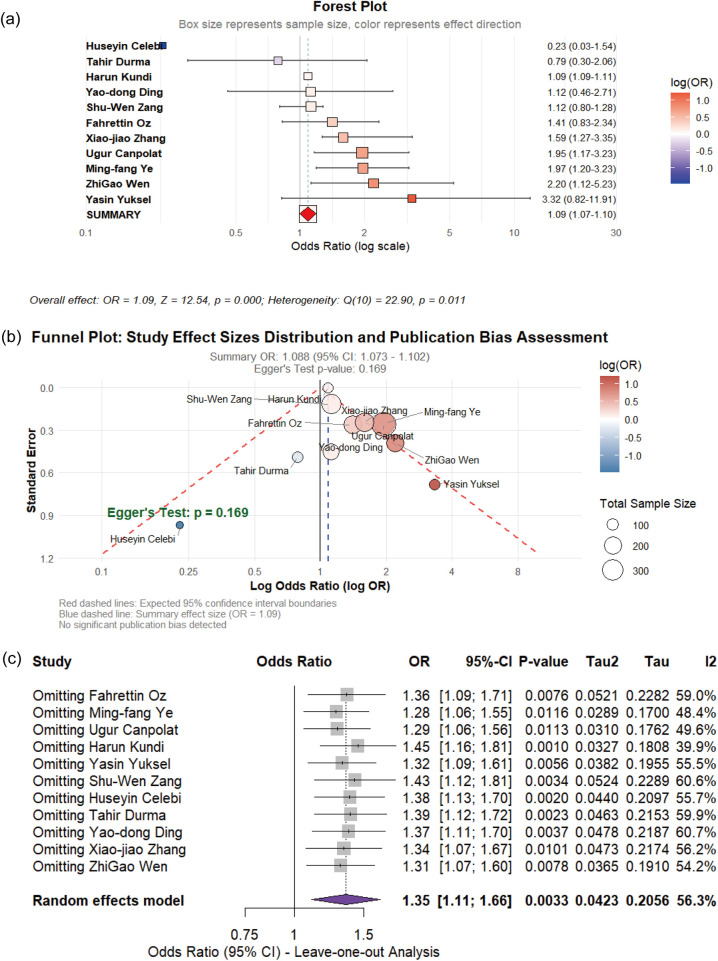
**(a)** Forest plot of the impact of smoke on CSFP; **(b)** the funel plot; **(c)** sensitivity analysis.

Eleven observational studies evaluated the association between tobacco smoking and CSFP risk. Substantial heterogeneity was observed across the included studies (I^2^ = 62.2%), necessitating the use of a random-effects model. The pooled analysis demonstrated a statistically significant positive association between smoking exposure and CSFP risk (OR = 1.09, 95% CI: 1.07–1.10, p < 0.01). Egger's regression test showed no significant publication bias (*p* = 0.169) (see Figure 8).

#### 3.3.4 Sensitivity analysis

The positive associations of WBC, PLR, BMI, PC, LDL, smoking with CSFP demonstrate high statistical robustness and are the most reliable findings of this study. Although hsCRP and TG show significant associations, their evidence strength is weakened due to publication bias (hsCRP) or the influence of high-weight studies on effect estimation (TG, TC). The initial significant associations of HDL, hypertension, and diabetes are severely weakened when they are simultaneously affected by publication bias and sensitivity analysis shows unstable results.

## 4 Conclusion

Our comprehensive meta-analysis ultimately revealed that several biochemical and clinical indicators demonstrate significant predictive value in assessing the potential risks of CSFP. The effect values (ORs, CIs) of each risk factor are summarized in Table 2. The pooled evidence identified WBC, Diabetes, TG, TC, PLR levels, hypertension status, current smoking, BMI and platelet counts as independent prognostic factors with clinical utility. These validated biomarkers collectively establish a multifactorial evaluation framework that enhances risk stratification and informs therapeutic decision-making in clinical practice. Notably, the potential prognostic significance of Hypertension, hsCRP, and HDL in CSFP pathogenesis remains inconclusive. These parameters necessitate further investigation through large-scale prospective cohort studies and mechanistic research to elucidate their pathophysiological relevance and establish evidence-based clinical application guidelines.

**Table 2:** Summary of effect sizes (ORs, CIs) for each risk factor in the meta-analysis.

prediction models	Risk factors	ORs	95% CIs	*P* value
circulating biomarkers	WBC	1.07	1.04–1.10	*p* < 0.01
	HsCRP	1.01	1.00–1.01	*p* < 0.01
	PLR	1.01	1.01–1.02	*p* < 0.01
	HDL	0.99	0.96–1.02	*P* = 0.54
	LDL	0.99	0.97–1.02	*p* = 0.25
	TC	1.008	1.001–1.015	*p* = 0.019
	TG	1.01	1.01–1.02	*p* < 0.01
	Platelet count	1.009	1.006–1.011	*p* < 0.01
clinical variables	Hypertension	0.97	0.94–1.00	*P* = 0.34
	DM	1.15	0.89–1.47	*p* = 0.283
	BMI	1.09	1.05–1.13	*p* < 0.01
	Current smoke	1.09	1.07–1.10	*p* < 0.01

## 5 Discussion

Coronary angiography for assessing slow flow does not require the use of pressure guidewires or vasodilators (such as adenosine); it can be calculated solely through routine coronary angiography, significantly simplifying the procedural workflow and reducing technical risks (38). Compared to IMR: angiography avoids guidewire manipulation and the requirement for high perfusion status, demonstrating comparable diagnostic accuracy (39). Compared to CFR/MRR: angiography is unaffected by epicardial vascular disease or hemodynamic fluctuations, more directly focusing on microvascular resistance assessment (40, 41). Due to the absence of additional equipment or medications, angiography reduces healthcare costs and is more suitable for promotion in resource-limited settings.

The pathological mechanism and prognosis of CSFP are still unknown. Anatomically, the volume of adipose tissue around the coronary arteries was significantly correlated with CSFP (42, 43). The causal relationship between the two remains unclear. From an electrophysiological perspective, the ventricular repolarization in CSFP patients is delayed (44), The frontal QRS-T angle is significantly higher in the CSFP patients (45). However, these ECG changes did not differentiate between CSFP and patients with ischemic cardiomyopathy. More echocardiographic results indicate that CSFP exists to varying degrees of left ventricular dysfunction (46), but its effect on right ventricular function is not clear (47, 48). The potential risks of CSFP reported in many studies, such as smoking, concurrent hypertension, diabetes, triglyceride glucose index, platelet to neutrophil ratio, etc. These reports are inconsistent. Therefore, this study applies meta-analysis to clarify the risk factors of CSFP and provide further exploration value for clinical research.

Analysis of inflammatory factors revealed statistically significant associations between CSFP and WBC(OR = 1.07), PLR(OR = 1.01), and HsCRP(OR = 1.01). Although recent studies have reported links between CSFP and multiple inflammatory markers—such as interleukin-6, neutrophils, and monocytes—and even inflammatory-related miRNAs have shown significant expression in CSFP (49, 50), our findings suggest that the predictive utility of inflammatory factors alone remains limited. We propose two potential explanations for this observation. First, inflammatory factors exhibit considerable variability influenced by various comorbid conditions, which may reduce their specificity and increase confounding in CSFP prediction when other underlying diseases are present. Second, elevation of a single inflammatory marker may be insufficient to induce CSFP; rather, synergistic interactions between plasma soluble adhesion molecules and inflammatory mediators could play a critical role in its pathogenesis (32, 51). Furthermore, significant publication bias and generally small sample sizes characterize existing research on HsCRP in this context. Therefore, the predictive capacity of inflammatory biomarkers for CSFP should be interpreted with caution.

Among indicators of dyslipidemia, both TC(OR = 1.008) and elevated TC (OR = 1.01,) demonstrated only modest associations with CSFP. These results align with prior meta-analyses highlighting the predictive role of the triglyceride-glucose index in CSFP (32, 52), further supporting the relevance of triglyceride metabolism. To our knowledge, no previous reports have specifically linked total cholesterol to CSFP; therefore, its predictive utility requires further validation and should be interpreted cautiously. Studies examining HDL in this context are notably affected by substantial publication bias. Although we applied appropriate statistical adjustments (trim-and-fill method) to address this issue, HDL alone showed no significant predictive value for CSFP. Existing literature often emphasizes the combined use of HDL with inflammatory markers, cholesterol, and other parameters for CSFP prediction (53, 54), a perspective consistent with our finding that HDL in isolation lacks independent predictive power.

Current smoking (OR = 1.09, 95% CI: 1.07–1.10) and platelet count PC(OR = 1.009) were identified as independent predictors of CSFP, whereas hemoglobin level demonstrated no significant prognostic association. The pro-thrombotic effects of smoking and heightened platelet activity may exacerbate endothelial dysfunction, implicating microvascular thrombo-inflammatory processes in the pathogenesis of CSFP (54, 55). Although hypertension initially exhibited a strong predictive association in the pooled analysis (OR: 1.38, 95% CI: 1.16–1.69), significant publication bias was detected. After applying trim-and-fill correction, the association was no longer statistically significant. Hypertension may contribute to CSFP via impaired coronary autoregulation, endothelial nitric oxide dysfunction, and vascular remodeling (56). While CSFP has been linked to left atrial enlargement and diastolic dysfunction (57, 58), the causal relationships among duration of hypertension, left ventricular structural changes (i.e., hypertensive heart disease), and CSFP progression remain unclear and warrant further detailed investigation.

Diabetes mellitus initially appeared predictive (OR = 1.16), but substantial publication bias was present. Following trim-and-fill adjustment, the pooled effect size was no longer significant (OR = 1.15, 95% CI: 0.89–1.47, *p* = 0.28), indicating no robust association between diabetes *per se* and CSFP. This finding does not contradict existing literature, as recent research has focused primarily on the role of elevated glycated hemoglobin and hyperglycemic states in CSFP (59, 60). Moreover, the impact of diabetes on vascular endothelial injury, adhesion molecules, and inflammatory mediators varies across disease stages (61), necessitating a more nuanced analysis of the diabetes-CSFP relationship.

BMI showed a consistent and significant association with CSFP (OR = 1.09, 95% CI: 1.05–1.13), aligning with prior evidence. Obesity may lead to dysregulated coronary blood flow, potentially mediated by potassium-channel dysfunction. Some studies suggest that mineralocorticoid receptor antagonists can mitigate obesity-related flow abnormalities by improving coronary microvascular function, such as reducing small-artery stiffness (62). Additionally, obese individuals often exhibit reduced coronary volume relative to myocardial mass, implying that structural remodeling of the microcirculation may alter hemodynamics (63). Concomitant metabolic disturbances in obesity, including insulin resistance, may further exacerbate CSFP (64).

Our study offers valuable clinical insights. Intensive smoking cessation support, weight reduction through diet and exercise interventions have been shown to lower the risk of CSFP and provide clear clinical benefits. These measures should therefore form the cornerstone of first-line intervention strategies. For CSFP patients with Dyslipidemia, early initiation of statin therapy is recommended. Statins not only lower lipid levels but also help stabilize endothelial function and slow the progression of subclinical atherosclerosis, extending their benefits beyond simple lipid modification.

Platelet count demonstrated stronger predictive value than PLR, implicating platelet hyperactivity in CSFP pathophysiology. This supports a potential role for antiplatelet agents (e.g., aspirin, clopidogrel), though clinical efficacy remains unconfirmed in randomized trials. For CSFP patients with high platelet counts or additional thrombotic risks, antiplatelet therapy may be considered after individual bleeding risk assessment. These patients should be prioritized for inclusion in future trials to establish definitive treatment efficacy.These suggestions are “hypotheses” generated based on correlational data, and the improvement in CSFP after intervention of the aforementioned factors still needs to be verified by more clinical trials (such as randomized controlled trials).

This study has several limitations. First, Most of the original studies included reported only unadjusted or insufficiently adjusted estimates of associations. Only 5 studies adjusted for known important confounders (age, gender, medication use, etc.) in their analysis. Therefore, the effect sizes summarized in this analysis primarily reflect the ‘reported associations’ from these insufficiently adjusted studies, rather than ‘independent associations’ or ‘causal effect estimates’ after strict confounding control. These unadjusted associations are likely to be influenced by residual or unknown confounding factors, which limits our ability to infer the independent causal relationship between exposure and outcome. Second, key parameters such as coronary artery diameter, the triglyceride-glucose index, and neutrophil-to-lymphocyte ratio were omitted due to insufficient comparable data, limiting the completeness of conclusions. Third, significant publication bias was noted for some indicators. While corrected via Trim-and-fill method, this indicates possible effect overestimation from small studies, necessitating further validation with large-scale clinical data.Future research should prioritize: (1) large-scale cohort studies using standardized adjustment models to improve reliability and comparability; (2) systematic collection of multidimensional data, including coronary imaging metrics and novel inflammatory-metabolic biomarkers, to support more comprehensive meta-analyses; and (3) expanded sample sizes through multicenter, prospective designs to reduce publication bias and strengthen validation of clinical associations. In summary, enhancing methodological rigor, data systematization, and study scale will be essential to consolidate the evidence in this field.

## Data Availability

The datasets presented in this study can be found in online repositories. The names of the repository/repositories and accession number(s) can be found in the article/Supplementary material.
