# Supplementary figures and images for: Coronary slow flow phenomenon: a meta-analysis of clinical risk predictors
Source: Front Cardiovasc Med. 2026 May 7;13:1656151. doi: 10.3389/fcvm.2026.1656151 (PMC13190535; doi:10.3389/fcvm.2026.1656151)

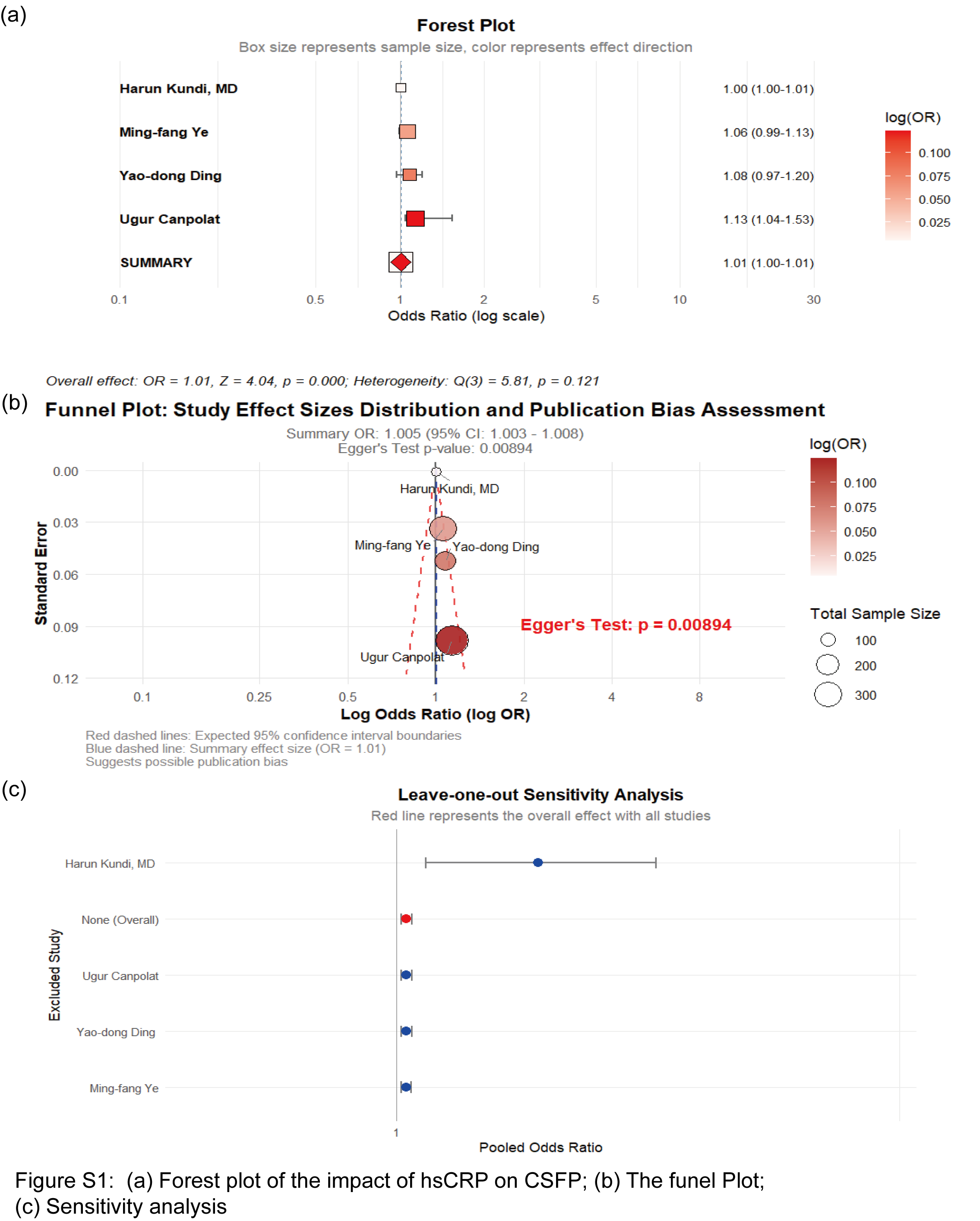

Supplement: Supplementary file 1 [file image1.tif]

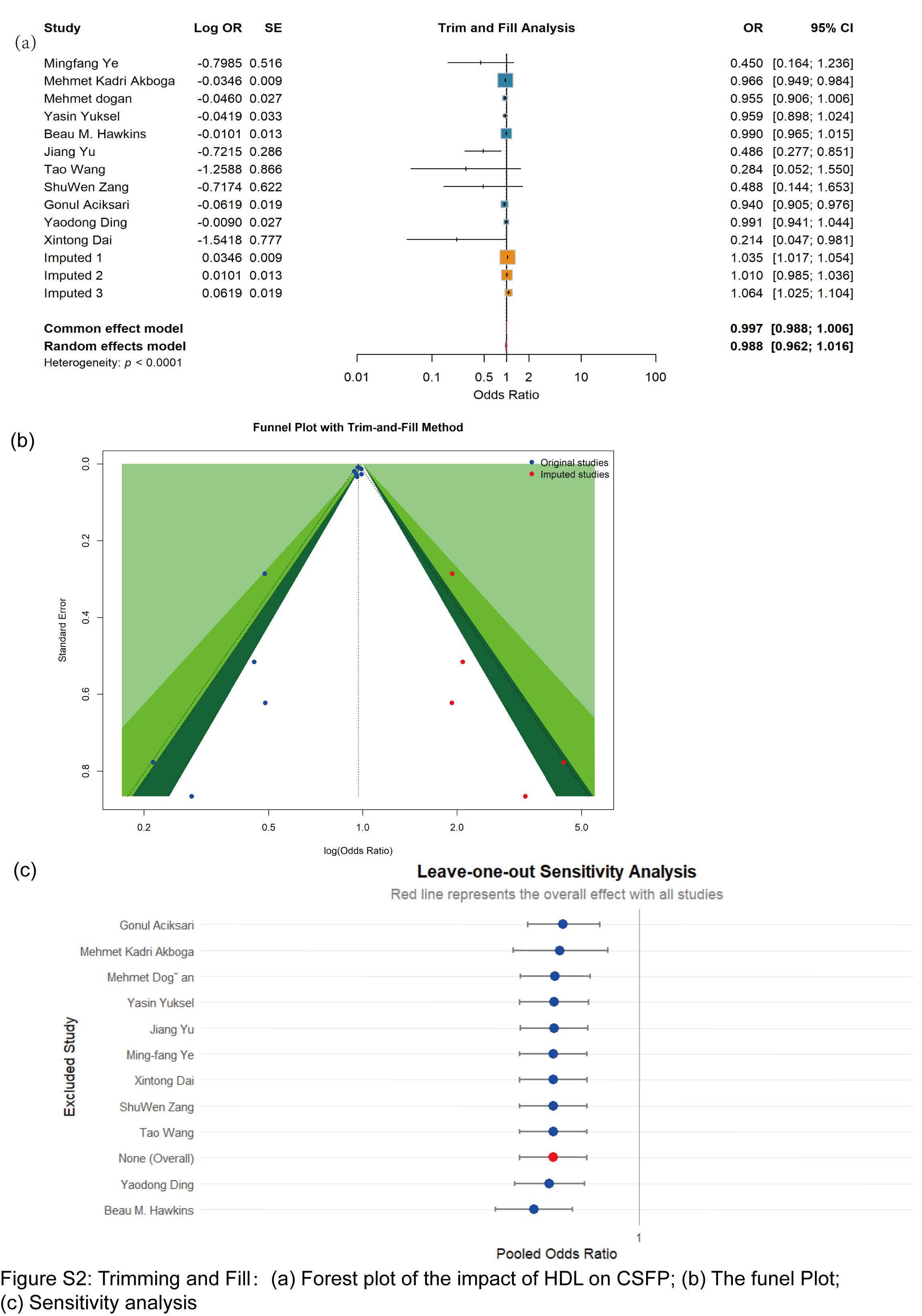

Supplement: Supplementary file 2 [file image2.tif]

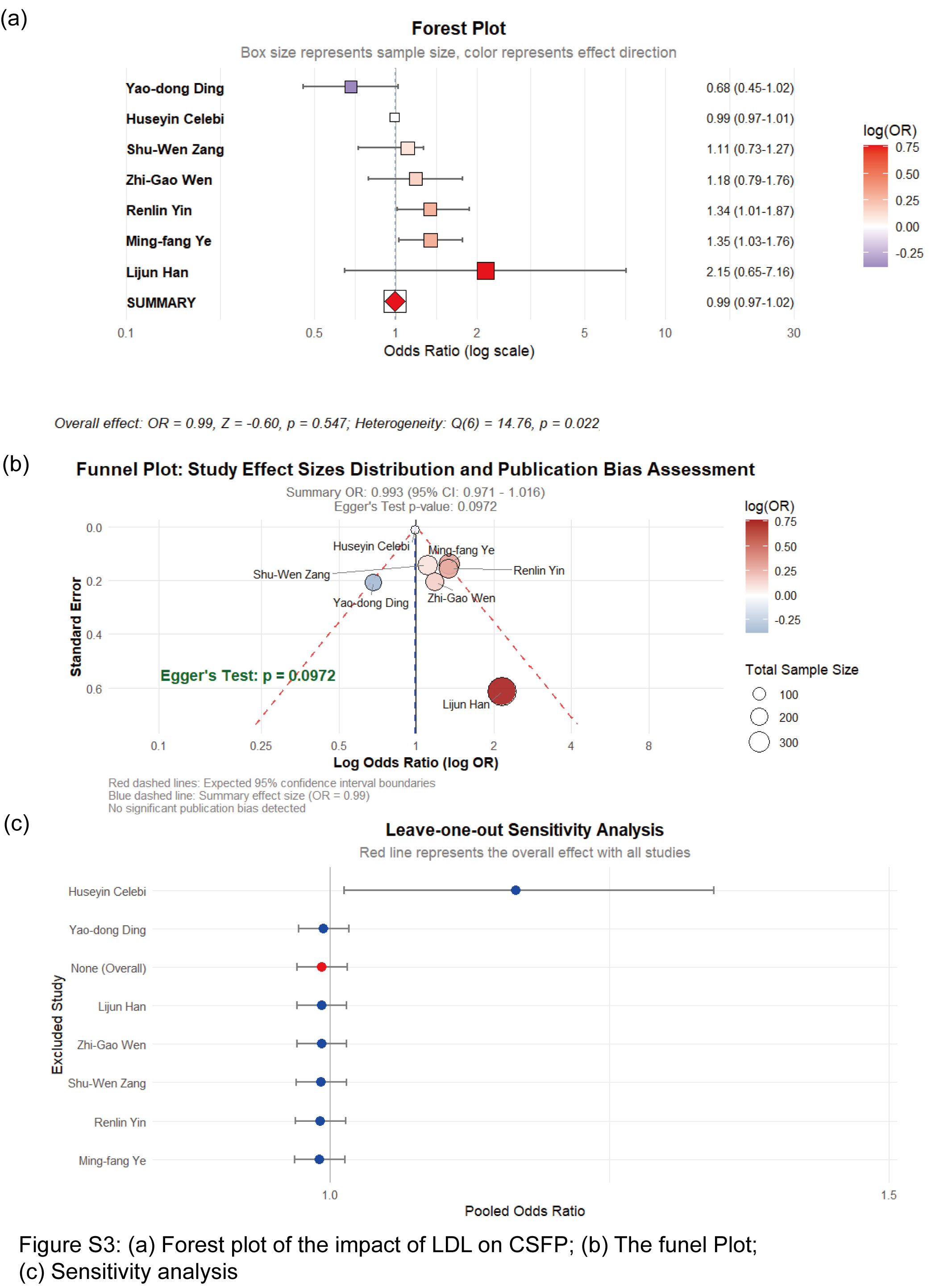

Supplement: Supplementary file 3 [file image3.tif]

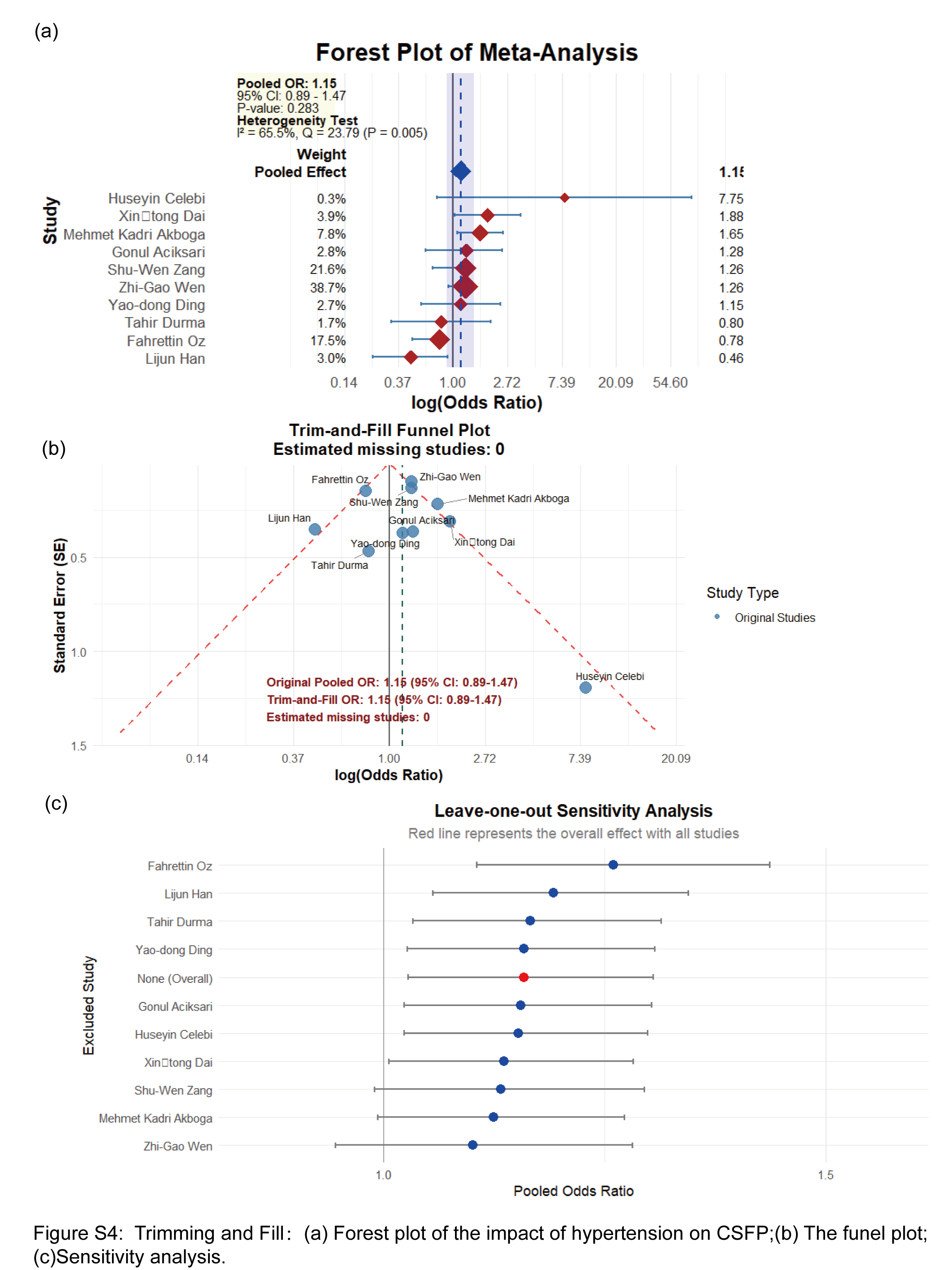

Supplement: Supplementary file 4 [file image4.tif]

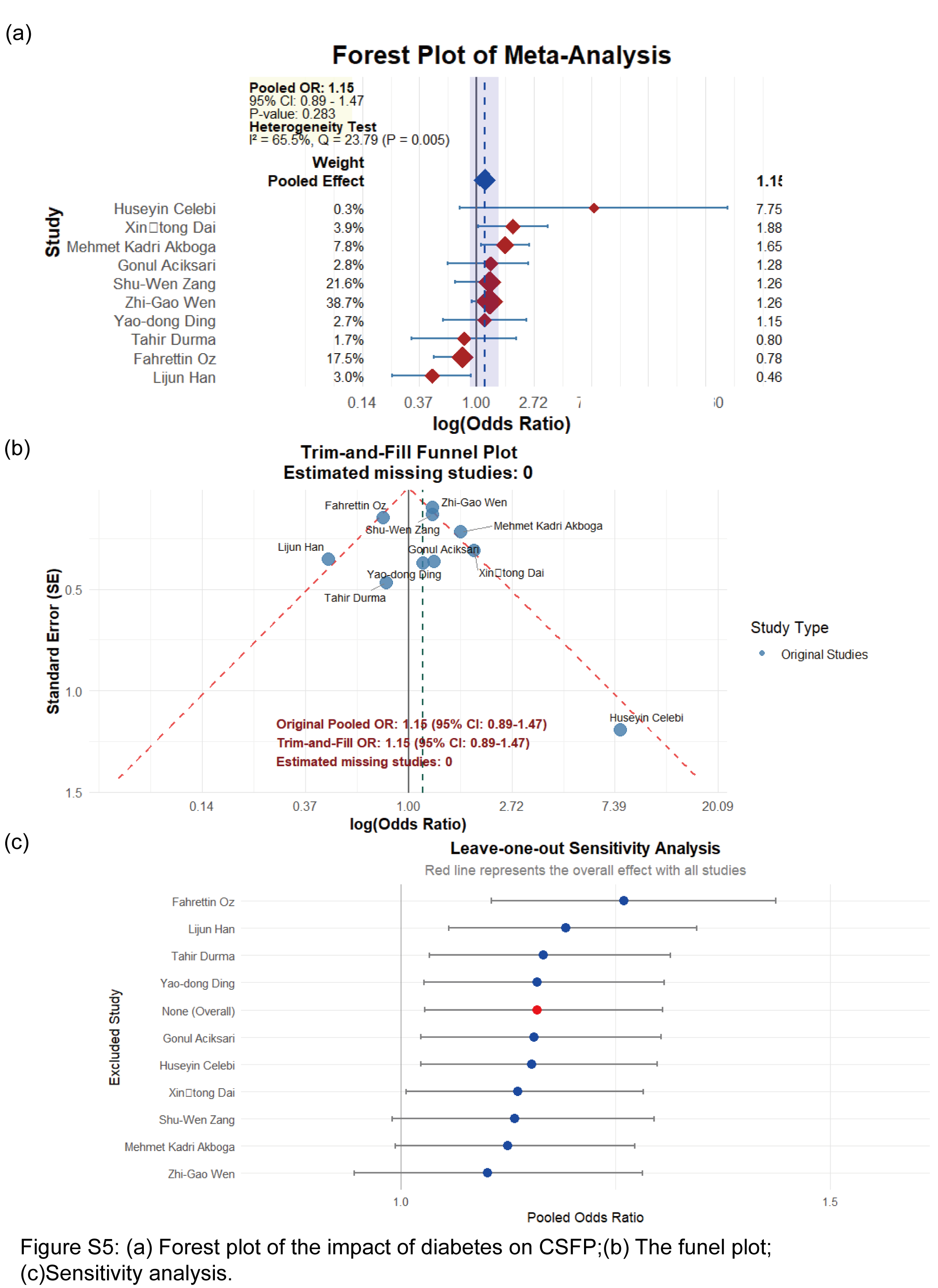

Supplement: Supplementary file 5 [file image5.tif]
